# Insights into Engineering Super-Duplex Stainless-Steel Microstructures: Composition Alterations and Processing Strategies in LPBF

**DOI:** 10.3390/ma19112344

**Published:** 2026-06-01

**Authors:** Leonidas Karavias, Leonidas Gargalis, Evangelia K. Karaxi, Elias P. Koumoulos

**Affiliations:** 1 Conify, Panteli Nikolaidi 23A, Agios Ioannis Rentis, 182 33 Athens, Greece; 2 IRES—Innovation in Research & Engineering Solutions, Silversquare Europe, Square de Meeûs 35, 1000 Brussels, Belgium

**Keywords:** LPBF, super-duplex stainless steel, austenite, microstructure, composition, additive manufacturing, processing strategies, laser engineering, hardness, nanoindentation

## Abstract

This study investigates in situ methodologies for enhancing austenite formation in Laser Powder Bed Fusion (LPBF)-processed Super Duplex Stainless Steel (SDSS), aiming to eliminate the requirement for post-process heat treatments. The evaluated approaches included layer remelting, increased layer thickness (from 40 μm to 80 μm), and chemical modification by blending SDSS with Stainless Steel SS316L at a 50/50 weight ratio. Microstructural characterization and macro-hardness testing were conducted, complemented by nanoindentation analyses to assess the local mechanical response of the austenite and ferrite phases in samples exhibiting the highest austenite content. The findings indicate that neither layer remelting nor increased layer thickness alone substantially elevated austenite content; the as-built microstructure remained predominantly ferritic under these conditions. In contrast, compositional adjustment through SS316L powder blending yielded a significant increase in austenite, resulting in a duplex microstructure. These compositional changes and the resulting phase balance were associated with a reduction in macro-hardness relative to the ferritic microstructures. Nanoindentation results showed comparable nanomechanical properties in both phases, suggesting that the decreased macro-hardness in the duplex microstructure is primarily attributable to changes in chemical composition and diminished solid-solution strengthening, rather than the increased austenite fraction itself. These results highlight the limitations of thermal strategies alone in achieving phase balance in LPBF-processed SDSS and demonstrate the effectiveness of compositional tuning in promoting favorable duplex microstructures.

## 1. Introduction

Super-duplex stainless steel (SDSS) is a class of engineering material renowned for its exceptional combination of high mechanical strength and outstanding corrosion resistance, attributed to a nearly balanced two-phase microstructure of ferrite (α) and austenite (γ) [1,2]. This unique combination of properties makes this alloy fit for demanding applications in the offshore, chemical processing, and oil and gas industries [2,3]. However, when this type of alloy is processed via Laser Powder Bed Fusion (LPBF) additive manufacturing (AM), it presents a predominantly ferritic microstructure with insufficient austenite content due to the intrinsic rapid solidification and high cooling rate in the manufacturing process [4,5,6]. This microstructure results in degraded ductility and toughness and compromised corrosion resistance, severely limiting the industrial adoption of as-built LPBF-processed SDSS [5,6,7,8].

Traditionally, the duplex microstructure derives from post-process heat treatments, such as high temperature (>1000 °C) annealing [7,9,10]. While effective, these heat treatments remove key AM advantages by introducing additional production steps, time, cost, and the risk of distorting geometrically complex parts. Consequently, the in situ austenite development, via innovative manufacturing strategies, can contribute to achieving a favorable phase balance during the build process itself.

Increasing the austenite fraction in LPBF-processed SDSS hinges on moderating the effective cooling rate during and between laser-induced thermal cycles. For SDSS, reducing scan speed lowers the cooling rate and can promote γ formation, yet the increase observed under practical build conditions is modest, and the usable parameter window is narrow when density and productivity constraints are considered [10]. Vanini et al. [11] found that, within practical process parameters required for high density, the increase in austenite content is limited. As scan speed decreases, issues such as increased porosity and reduced productivity arise. Their study indicated that while scan speed adjustments can influence thermal history, the range for effective modification narrows when considering the gas atmosphere and densification requirements, resulting in only a minor increase in austenite compared to the dominant ferrite phase typical of as-built SDSS. This aligns with broader findings for SDSS, where optimizing laser power, scan speed, and hatch distance produces nearly fully dense components, but the microstructure remains largely ferritic—for example, approximately 89% ferrite in grade 2507 (SDSS) at optimal settings. These results highlight that scan speed modification alone rarely achieves a balanced duplex phase fraction and often requires additional strategies, such as thermal or compositional adjustments, to promote significant austenite formation.

Beyond scan speed, layer thickness (t) directly modulates the interlayer thermal history in SDSS via the volumetric energy density relationship [12]; an increased layer thickness lowers the effective cooling rate and extends the dwell time between successive laser exposure conditions that, in principle, favor γ-austenite nucleation and growth. In 2507 SDSS, parameter optimization studies acknowledge the influence of layer thickness, but typically it is held constant while varying laser power, scan speed, and hatch distance; under such processing conditions, the as-built microstructure remains predominantly δ-ferritic, indicating that conventional laser power–scan speed–hatch spacing tuning alone rarely restores duplex balance [11]. Industrial process documentation for SDSS powder with varying chemical compositions further qualifies 40/80 μm layer-thickness strategies [13], which increase the volumetric build rate and alter the temporal thermal profile per layer in a manner consistent with thicker-layer cooling histories and an increased austenite phase fraction. Dadbakash et al. [14] investigated LPBF tool steel using 20 μm and 40 μm layer thickness and found that the thicker layers led to lower residual stress and more retained austenite, attributed to the reduced cooling rates; although the alloy differs from SDSS, the mechanism-extended thermal dwell and reheating of underlying material is directly transferable to duplex stainless-steel grades.

A second route is to engineer cyclic reheating by applying laser remelting per-layer. In AlSi10Mg, layer-by-layer remelting improved surface quality and hardness and refined the solidification microstructure, consistent with accelerated solidification of the remelted melt pools and repeated thermal cycling of the underlying material [15]. Related studies on Ti-6Al-4V and Ti–Zr β-titanium alloys likewise report microstructural modification and property gains after laser remelting, typically linked to defect mitigation and substructure refinement [16,17,18]. However, alloy sensitivity is evident in 18Ni-300 maraging steel, where remelting slightly coarsened the average grain size and promoted nanoprecipitation through the added heat cycles, illustrating that reheating can shift strengthening mechanisms without guaranteeing grain refinement [18]. Remelting can also be combined with hatch-distance manipulation to tune morphology: pairing layer remelting with reduced hatch spacing altered grain aspect ratios by rotating grains, without necessarily decreasing grain size, demonstrating that lateral overlap and re-exposure can reorient the solidification texture independently of coarsening/refinement trends [19]. In Duplex Stainless Steel (DSS) (22Cr and Ni-over-alloyed), repeated remelting cycles homogenized the austenite distribution and solute dispersion without increasing the bulk γ fraction (~35%), while modestly improving the critical pitting potential and critical pitting temperature; at the same time, the remelted condition showed greater localized corrosion-volume loss, indicating that remelting can lower pit nucleation propensity yet accelerate pit growth—i.e., opposing effects on corrosion performance driven by microstructural redistribution rather than phase-fraction change. This indicates that such contrasting effects on corrosion performance are primarily influenced by microstructural redistribution rather than changes in phase fraction [20]. Evidence shows that per-layer remelting in LPBF can adjust defect levels, crystallographic texture, and the local electrochemical response. However, no published studies have explored its effect on increasing γ-austenite in as-built SDSS microstructures.

Beyond thermal-history control, a complementary route to increase austenite is chemical tuning. Raising the content of austenite γ-stabilizers (notably Ni and Mn, together with N where applicable) in the SDSS composition promotes the δ-ferrite→γ-austenite transformation during solidification and cyclic reheating [21,22]. In situ chemical modification of SDSS to increase and stabilize γ-austenite can be achieved by powder blending/in situ alloying, either mixing SDSS with austenitic SS316L in controlled proportions or dosing elemental Ni to enrich γ-stabilizers during melting. SDSS/DSS–316L blends have demonstrated a clear rise in the as-built γ fraction under LPBF, with the phase balance shifting as the 316L proportion increases [23,24,25,26,27], providing a practical route to influence microstructure. Complementarily, Ni over-alloying via powder mixing [27] has been used to elevate γ in LPBF-processed duplex grades; however, its effects on the mechanical response and pitting resistance depend on subsequent heat treatments and the resulting phase morphology, motivating careful coupling of composition tuning with thermal-history control. Achieving chemical homogeneity with elemental additions requires sufficient melt-pool dwell time and energy input to promote mixing and limit segregation. The operating window is further conditioned by powder properties (size distribution, flowability, and absorptance), which shift the boundaries of conduction, transition, and keyhole melting regimes.

In contrast to the prevailing reliance on post-build solution annealing to restore phase balance in LPBF-processed SDSS, which adds time and additional processing steps, the present work interrogates three in-process levers that deliberately steer thermal history and chemistry toward γ-austenite stabilization: (i) increasing layer thickness from 40 to 80 μm to reduce interlayer cooling rates and extend residence within the 800–1200 °C nucleation window; (ii) per-layer rescanning/remelting to modulate cyclic reheating of underlying material, and thus alter phase-transformation kinetics and residual stress evolution; and (iii) powder-chemistry tuning via a 50/50 SDSS–316L blend to enrich γ-stabilizing elements and shift the as-built phase balance, in line with established welding practice, where over-alloyed Ni-rich fillers are routinely selected for DSS/SDSS joints to secure a duplex weld metal in the as-deposited condition [28]. The impact of each strategy on the microstructure is quantified by scanning-electron-microscopy and optical-microscopy image analysis of the γ-phase fraction, while macro-hardness testing captures the net mechanical response; nanoindentation mapping of a 50/50 γ/δ duplex condition resolves phase-separated nanomechanical properties and their contribution to bulk hardness, building on prior Electron Backscatter Diffraction (EBSD)-coupled nanoindentation protocols for duplex stainless steels and their application to LPBF systems.

Through this experimental design, the work advances the understanding of microstructure control during LPBF fabrication—aiming to reduce reliance on post-build heat treatments—and provides insights into microstructure engineering during laser processing of SDSS alloys.

## 2. Materials and Methods

### 2.1. Feedstock Materials and Powder Blending

**Figure 1:**
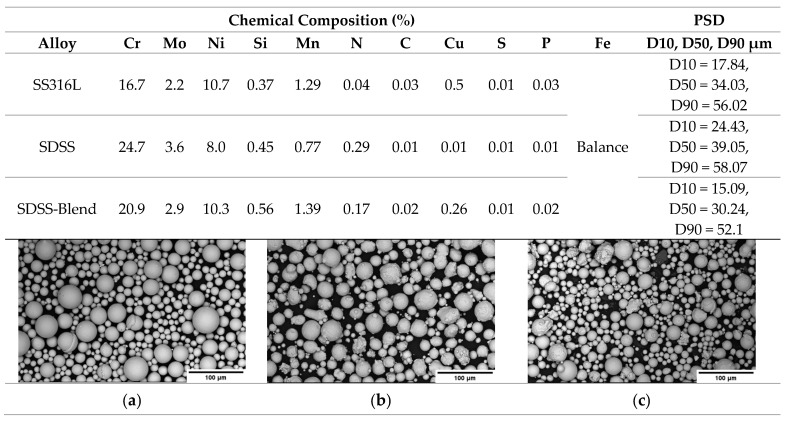
Chemical composition, PSD of SS316L, SDSS, and SDSS-Blend, and SEM images of (**a**) SS316L, (**b**) SDSS, and (**c**) SDSS-Blend powders showing their morphology.

Gas-atomized super-duplex stainless-steel (SDSS) grade 2507 (1.4462) (MARS F53, Mimete S.r.l., Biassono, Italy) and stainless-steel SS316L (1.4404) (Metalpine 316L, Metalpine GmbH, Graz, Austria) powders were utilized in this study. Alloy blending was performed between SDSS and SS316L in a 50/50 weight ratio to produce a custom/tailored SDSS-Blend. The powder blending process was conducted using a tumbling mixer operating at approximately 30 rpm for 15 min. In Figure 1, Particle Size Distribution (PSD), chemical composition, and powder morphology are presented for the SDSS, SS316L, and SDSS-Blend powders. The nominal chemical composition of the SDSS-Blend was theoretically calculated from the chemical compositions of SDSS and SS316L. The morphology of the powders is depicted through scanning electron microscopy (SEM) images, which were acquired utilizing a Phenom ProX (Thermo Fisher Scientific Inc., Waltham, MA, USA) SEM with the use of 15 kV acceleration voltage. The PSD of each powder was measured from 120 SEM micrographs, with each micrograph acquired at a 500 μm Field of View (FOV) using add-on image analysis software (Phenom ParticleMetric, V1.2.2.0) from Thermo Fisher Scientific, Waltham, MA, USA.

### 2.2. Processing Strategies and Parameter Optimization

**Figure 2:**
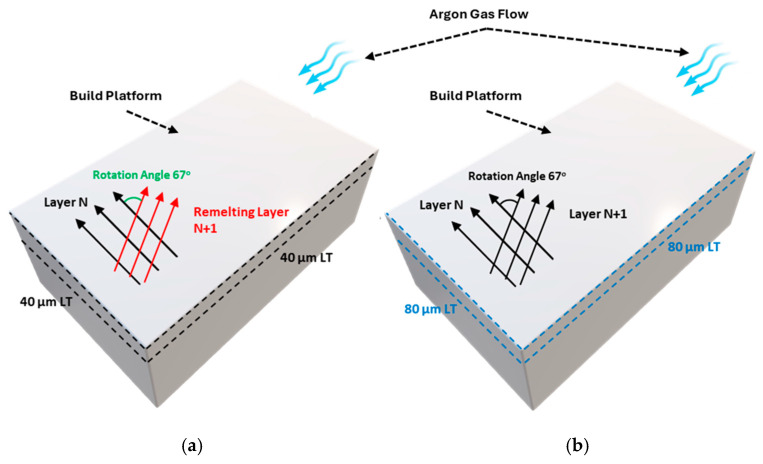
Graphical representations of the applied processing strategies on SDSS: (**a**) solidified layer remelting, (**b**) increased layer thickness from 40 μm to 80 μm. LT = layer thickness; N = the previous layer; N + 1 = the following layer.

In this study, a Laser Powder Bed Fusion (LPBF) system (INTECH, SF1 iFusion150, Intech Additive Solutions Ltd., Bangalore, India) equipped with a 500 W Ytterbium fiber laser (1064 nm) and laser spot size of 80 μm was used. The different processing strategies on SDSS powder alloy included solidified layer remelting and an increased layer thickness (LT) from 40 μm to 80 μm. Both strategies were utilized to promote austenite formation in the as-built microstructure of SDSS. By remelting the solidified layer, which results from the previous laser scan, a higher heat input was applied in the printed structure and, in turn, underlying areas were reheated to induce austenite nucleation. Essentially, each deposited powder layer underwent two laser scans: the first scan melted the deposited powder, while the second scan directly remelted the previously solidified layer without additional powder. The second processing strategy was to increase the layer thickness from 40 μm to 80 μm. The higher layer thickness was utilized to melt larger amounts of powder after each laser scan, resulting in larger melt pools. This also contributed to minimizing the cooling rate [29] and promoting austenite formation. A representative schematic of the two processing strategies is shown in Figure 2. One Design of Experiment (DoE) for each processing strategy was prepared and utilized based on the optimum parameters for SDSS alloys produced via LPBF with a 40 μm layer thickness, derived from previous work [10]. Additionally, the predefined optimum process parameters (>99.9% relative density) [10] for SDSS alloys with a 40 μm layer thickness were utilized to produce a reference sample. For the custom SDSS-Blend alloy, a separate DoE was prepared and used. A process-parameter-optimization study was implemented in both SDSS powder and SDSS-Blend. To determine the optimal process parameter set for each alloy and processing strategy, volumetric energy density (VED) was utilized as the primary design parameter. VED was calculated using Equation (1):

(1) $$E=\frac{P}{v\cdot h\cdot t}$$

where P represents the laser power in W; v is the laser scan speed in mm/s; h is the hatch distance between adjacent laser-scan tracks in mm; and t or LT is the layer thickness of the powder in mm. It must be noted that the varying energy densities for each DoE were a result mostly of laser power and scan speed. The layer thickness was the only parameter kept constant at 0.04 mm or 40 μm for the DoE of SDSS-Blend and the DoE of SDSS by applying the remelting strategy (SDSS-Remelting). For the DoE of SDSS with increased layer thickness (SDSS 80 μm LT), the value increased to 0.08 mm or 80 μm. The hatch distance was maintained at 0.1 mm for both SDSS-Remelting DoE and SDSS-Blend DoE, while it varied in SDSS 80 μm LT DoE. The layer scan rotation angle was set to 67° between successive layers for all DoEs. The set of parameters for each DoE is presented in Table 1, Table 2, and Table 3, respectively. In Table 1, the VED for each scan was calculated separately: Scan 1 (melting with powder) and Scan 2 (remelting without powder). Furthermore, a total VED—representing the combined contribution of both scans—was considered to ensure comparability with the other DoEs. Cubic samples of 10 × 10 × 10 mm (L × W × H) were built on 3 mm support structures to enable easier removal from the platform. The build platform was made of AISI 304 stainless steel with a diameter of 150 mm, and it was preheated to 150 °C during the printing process. Additionally, argon inert atmosphere (grade 5, 99.9% pure) was maintained throughout the printing process to prevent oxidation by keeping the oxygen levels below 0.05%.

### 2.3. Microstructural Characterization of LPBF Samples

Metallographic preparation was carried out for all samples to evaluate their relative density and microstructure. This procedure included cross-sectioning, using a precision micro-cutting machine (Mecatome T210, PRESI, Paris, France), mounting, grinding, and polishing (Tegramin, STRUERS, Birmensdorf, Switzerland). Relative-density measurements were obtained from cross-sectional optical microscopy (OM) images in the plane transverse to the build direction (XY) and the plane parallel to the build direction (XZ) (5 optical images per plane, i.e., 10 images per sample) using ImageJ (FIJI) software (version 1.54d, NIH and LOCI, Maryland and Wisconsin, USA). For microstructural analysis, the same metallographic procedure was followed, with an additional etching step. Each specimen was ground and mirror-like polished with a 1 μm diamond solution. The optimum polished samples were chemically etched for 5–10 s using Beraha’s reagent (80 mL of H_2_O, 40 mL of HCl, and 1 g of potassium metabisulfite) to enhance the phase contrast between austenite and ferrite and effectively reveal the microstructure. AMScope Light Optical Trinocular Metallurgical Microscope with Polarization (40–800×), equipped with an 18 MP Digital Camera (AMScope, United Scope LLC, Irvine, CA, USA), was used for capturing images for relative-density measurements (50× magnifications) and phase quantification (100× magnifications). Phase quantification was performed using optical microscopy (OM) micrographs, with analyses restricted to specimens exhibiting an austenite phase fraction of ≥30%. This restriction was adopted because Beraha etching colors austenite white and ferrite darker, and in predominantly ferritic microstructures with very thin austenite regions, the contrast was insufficient for reliable phase quantification with image analysis. Therefore, entirely ferritic microstructures were evaluated qualitatively. For each plane, five micrographs were acquired to ensure a representative dataset, and image analysis was carried out using ImageJ. Scanning electron microscopy (SEM) (Thermo Fisher Scientific, Phenom ProX, Waltham, MA, USA) was utilized for higher-magnification imaging of microstructural features. Energy Dispersive X-ray Spectroscopy (EDS) was utilized for the compositional analysis and identification of microstructural phases and constituents.

### 2.4. Mechanical Characterization of LPBF Samples

**Figure 3:**
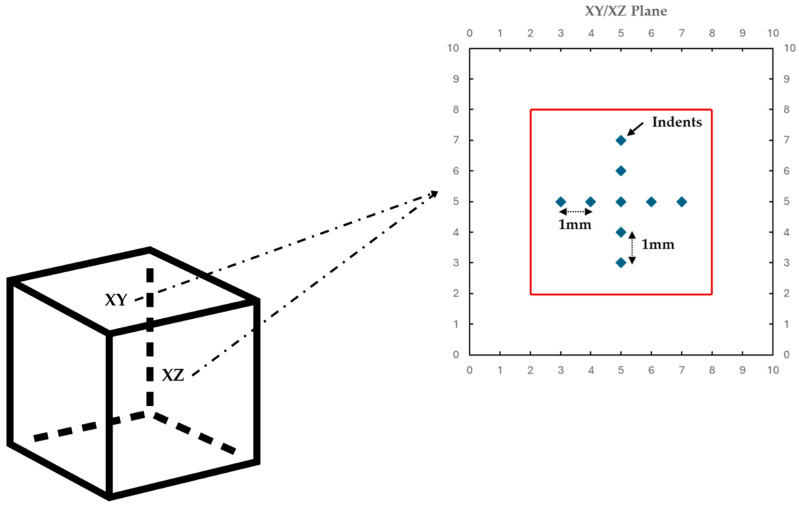
Illustration of the macro-hardness cross-pattern indentation in both XY and XZ planes. The red square indicates the measurable area, excluding 2 mm from the edges on each side.

Vickers macro-hardness measurements were conducted on the optimum as-built samples after the metallographic preparation using the Innovatest Falcon 400G2 Vickers micro/macro-hardness tester (INNOVATEST Europe BV, AA Maastricht, The Netherlands). The tests were performed at room temperature with a load of 10 kgf. Vickers macro-hardness measurements were performed on both XY and XZ planes, with the average value (per plane) calculated from 9 indents arranged in a cross pattern with intervals of 1 mm between them. The indent spacing was greater than the minimum distance specified in ISO 6507-1:2023 [30]. Furthermore, a 2 mm distance from the edges of the surfaces was kept to minimize the influence of edge-related anomalies, thus providing more reliable and representative hardness values. Edges often exhibit different surface finishes, work hardening, or oxidation compared to the bulk material. These edge-specific conditions can distort the indentation response, leading to inaccurate hardness measurements [31]. An illustration of the cross pattern is demonstrated in Figure 3. Further investigation of the phase-separated mechanical properties on the nano-scale was performed by implementing nanoindentation testing. The nanoindentation testing was applied on the sample that presented the highest austenite content and/or duplex microstructure. The sample was polished to a 0.04 μm finish using colloidal silica prior to testing. Measurements were conducted using a nanoindenter (Hysitron TS 77 Select, Bruker, Minneapolis, MN, USA). The instrument capabilities enable loading from 1 × 10^−3^ mN to 30 mN, with a high load and displacement resolution of 1 nN and 0.04 nm, respectively. The instrument is equipped with a scanning probe microscope, in which a probe tip moves in a scan pattern across the sample surface using a three-axis piezo positioner. For this, a Berkovich tip of 80 nm radius was selected. Nanomechanical properties were assessed using the Oliver–Pharr model [32]. A grid mapping protocol of a 20-by-20-point array was selected, with a spacing of 10 microns, employing 40 s in loading, a 3 s hold time, and a 40 s unloading time under displacement control (set at 200 nm of displacement). The load range was carefully selected according to [33], where values of reduced modulus were stable over 3000–7000 μN. A methodology similar to that reported in a previous study [26] was employed to match the indents with the corresponding identified phases, followed by a manual annotation process. Phase-resolved nanoindentation was performed using grid mapping coupled with Electron Backscatter Diffraction (EBSD)-based phase labeling. Indents intersecting grain boundaries (as identified by EBSD) were excluded from analysis. The nanomechanical properties were calculated, namely the reduced modulus E_r_ (GPa), hardness H (GPa), normalized pile-up-to-sink-in height (h_c_/h_max_), and the plasticity index, which is expressed through the H/E_r_ ratio.

Additionally, the area under the load–displacement curve (AUC) was used as a measure of indentation work and energy absorption. Specifically, three energy components were calculated. The loading AUC represents the plastic work input during indentation and corresponds to the area under the loading curve. The unloading AUC reflects the elastic recovery energy and was computed as the area under the unloading curve, taken in absolute value. The total AUC represents the total work (plastic and elastic) and is the total area under the loading and unloading curves. The areas under the loading and unloading curves are calculated through integration. A schematic representation of the load–displacement curve, along with the corresponding loading and unloading areas, is shown in [34].

The AUC values were computed numerically using the trapezoidal integration method implemented via the NumPy library [35] in Python (NumPy, V2.0.0). This approach enabled accurate estimation of energy values from the discrete load–displacement (P–h) data points collected during each indent.

Several indentation curves exhibited pop-in events, which are sudden displacement bursts typically occurring in the early stages of loading. To mitigate their influence on energy calculations, two parallel data-processing strategies were employed. In the first approach, entire indentation curves exhibiting pop-ins were excluded from the dataset. In the second approach, the pop-in region was manually identified and removed from the loading curve, after which the AUC was computed for the remaining portion of the curve. Both approaches were applied independently to assess the robustness of the energy metrics and to evaluate the sensitivity of the results to early-stage deformation anomalies.

For each phase and each processing approach (direct and truncated), statistical descriptors were calculated for the loading AUC, unloading AUC, and total AUC. These included the count of valid indents, mean, and standard deviation. The statistics were reported in nominal energy units (picojoules, pJ) and, where appropriate, as percentages relative to the mean. The data were grouped by phase label (ferrite and austenite) based on microstructural identification, and the results were used to compare the deformation and energy absorption characteristics of each phase.

## 3. Results

### 3.1. Relative Density

**Figure 4:**
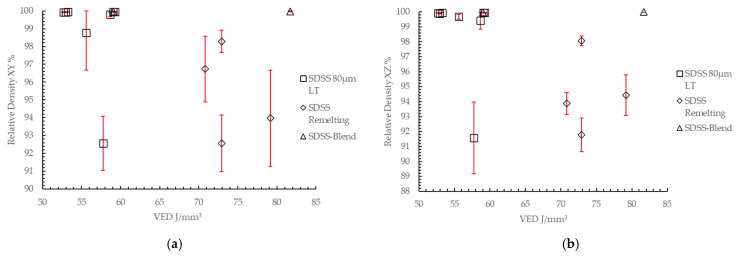
Relative density of (**a**) XY planes and (**b**) XZ planes as a function of VEDs for all DoEs and their as-built samples.

In Figure 4, relative density results as a function of the calculated Volumetric Energy Densities (VEDs) are presented along with the optimum set of parameters for each Design of Experiment (DoE), corresponding to the highest-density samples. The remelting-strategy DoE (SDSS-Remelting) resulted in relative densities up to 98.3% and 98.1% in the XY and XZ planes, respectively, at a total VED value of 73 J/mm^3^ (RM_1 sample). The 80 μm layer-thickness DoE (SDSS 80 μm LT) presented the majority of samples with relative densities > 99%, with the optimum samples reaching values of >99.9% in both XY and XZ planes, obtained at 53 and 59 J/mm^3^, respectively. The 80 μm_1 sample produced with a VED value of 53 J/mm^3^ resulted in slightly higher relative density (99.94% XY, 99.96% XZ) in comparison to the 80 μm_3 sample produced with 59 J/mm^3^ (99.94% XY, 99.93% XZ), and it was selected as the optimum from the 80 μm layer-thickness DoE.

A low and high VED value of 59 and 82 J/mm^3^, respectively, resulted in high-density samples exceeding 99.9% relative density for the powder blend. The high-VED (82 J/mm^3^) sample (SDSS-Blend_2) was slightly better in terms of relative density (99.96% in the XY plane and 99.99% in the XZ plane) and was considered the optimum for further characterization.

**Figure 5:**
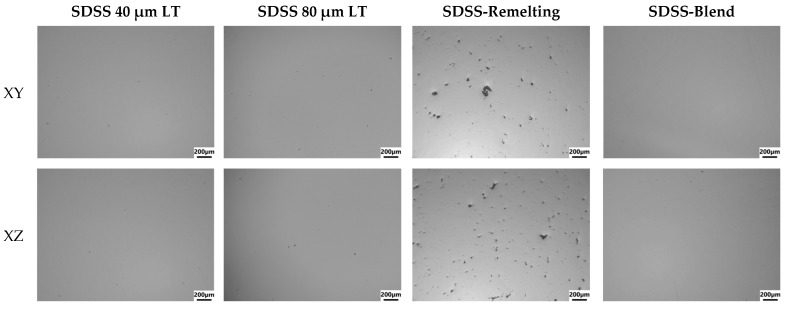
Optical micrographs of the optimum relative-density sample from each DoE in both XY and XZ planes.

In Figure 5, the optical micrographs of the optimum samples for both XY and XZ planes from each DoE are presented. In the SDSS 80 µm LT and SDSS-Blend specimens, both the XY and XZ build planes were dominated by small, near-spherical pores. This morphology is characteristic of gas porosity rather than lack-of-fusion (LoF) or keyhole porosity, which typically manifests as larger, faceted cavities aligned with hatch or interlayer interfaces [36]. The absence of LoF in these conditions indicates sufficient track overlap and melt-pool penetration, yielding continuous bonding across adjacent scan tracks and layers [37]. By contrast, the remelting condition (SDSS-Remelting) exhibited planar, boundary-parallel voids consistent with LoF. Specifically, the RM_1 sample (total VED = 73 J/mm^3^) was dominated by LoF porosity in both the XY and XZ planes, as revealed by optical micrographs. Only a minor population of round, isolated pores was observed, consistent with entrapped-gas or keyhole-type porosity, and was negligible compared to LoF.

### 3.2. Microstructure

**Figure 6:**
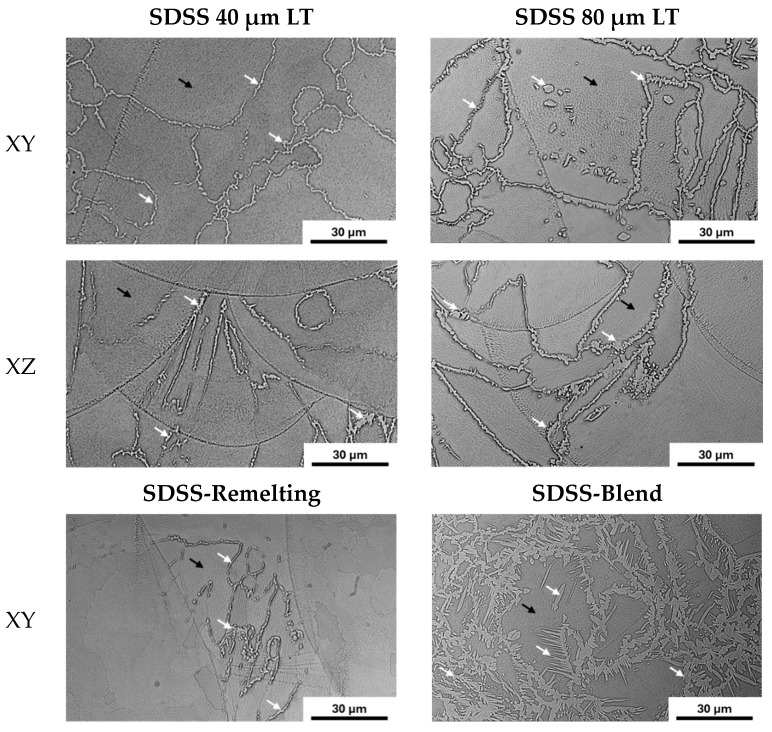

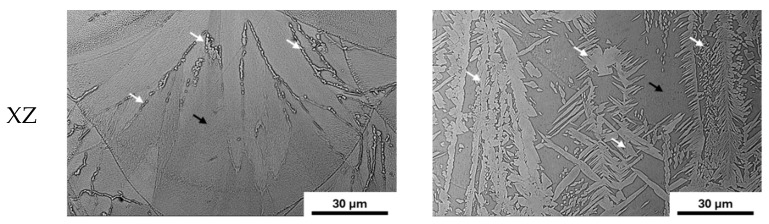
Microstructures of SDSS 40 μm LT, SDSS 80 μm LT, SDSS-Remelting, SDSS-Blend. Austenite is indicated by white arrows, while ferrite is indicated by black arrows.

**Figure 7:**
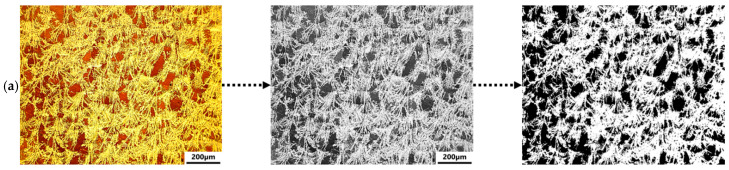

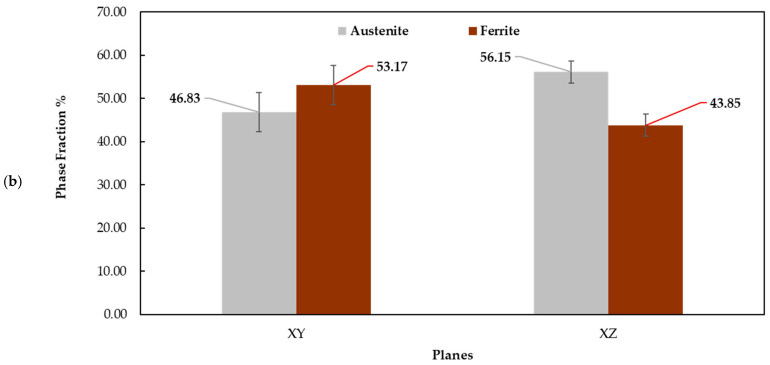
Image analysis results of the SDSS-Blend: (**a**) representative optical micrograph of the XZ plane, converted to grayscale and subsequently thresholded for phase quantification, (**b**) column chart illustrating the phase fractions of austenite and ferrite.

The microstructures of the optimum samples are shown in Figure 6. The microstructures of the SDSS 40 μm LT, SDSS 80 μm LT, and SDSS-Remelting samples revealed only limited amounts of grain boundary austenite (GBA), which nucleated along the ferrite grain boundaries [38]. In the SDSS 80 μm LT microstructure, GBA, which appeared coarser, nucleated at the majority of ferrite grain boundaries. The SDSS-Blend sample illustrated a δ-ferrite matrix with two clear austenite forms: thin and coarse films along ferrite grain boundaries and fine, plate-like laths that grow into the ferrite grains. The plates appear in aligned packets, with branching and impingement, which is typical of Widmanstätten-type austenite rather than coarse, equiaxed γ-austenite [21,39]. Phase identification via Energy Dispersive X-Ray Spectroscopy (EDS) confirmed the presence of austenite and ferrite in both alloys (SDSS and SDSS-Blend), irrespective of the processing conditions. Representative chemical compositions of each phase are summarized in Table 4. The principal difference between the two phases was identified in Mo concentration, which was consistently 1–2 wt% higher in ferrite compared to austenite, reflecting its role as a ferrite stabilizer [40]. The other chemical elements showed almost similar chemical concentrations between the austenite and ferrite phases. The SDSS 40 μm LT and SDSS 80 μm LT samples revealed similar austenite/ferrite compositions, whereas the SDSS-Remelting microstructure featured altered elemental partitioning, with higher Mo and lower Cr contents in both phases compared to the 40 μm and 80 μm layer-thickness conditions. For the SDSS-Blend, both phases contained reduced alloying element concentrations relative to the SDSS samples, as expected due to the modified nominal composition. Figure 7 presents an optical micrograph of the SDSS-Blend microstructure, alongside its segmented image used for quantitative analysis and the image-analysis results as well. The austenite content, measured via automated image analysis, was approximately 47% and 56% in the XY plane and XZ plane, respectively, indicating a phase balance between austenite and ferrite was achieved in the SDSS-Blend alloy. The observed difference in austenite content between the XY and XZ planes arises from the microstructural distribution: in the XZ plane, austenite grains frequently extend across multiple melt pools due to the build direction and thermal gradients, whereas in the XY plane, they are more confined within individual melt pools. This orientation-dependent distribution affects threshold segmentation during image analysis and results in different measured austenite fractions between the planes. Similar build-orientation-dependent phase and microstructural variations have been reported in Laser Powder Bed Fusion (LPBF) duplex stainless steels, where Electron Backscatter Diffraction (EBSD) phase maps showed differing austenite contents in the XY and XZ sections of the same build [26].

### 3.3. Macro-Hardness

**Figure 8:**
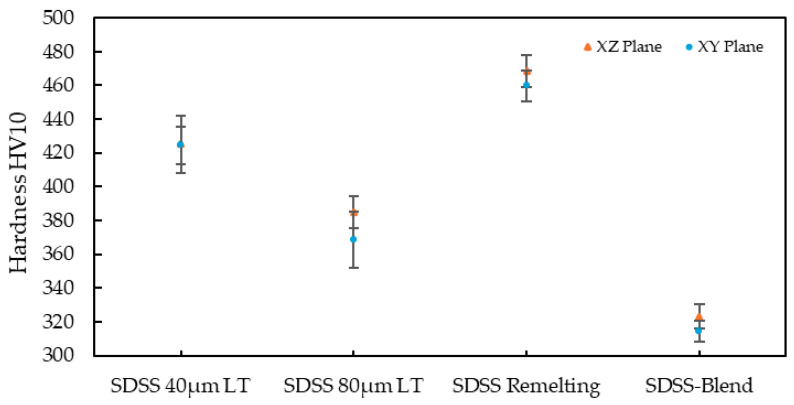
Vickers hardness (HV10) variation between alloys and conditions.

The average Vickers macro-hardness (HV10) values for each alloy, condition, and plane are summarized in Table 5, along with their standard deviations. Additionally, a diagram illustrating the variation in hardness across different alloys and conditions is presented in Figure 8. The macro-hardness results demonstrate that the SDSS-Remelting sample exhibited the highest hardness, with mean values of 459.73 HV10 in the XY plane and 468.57 HV10 in the XZ plane. In contrast, the SDSS-Blend sample presents the lowest hardness, with mean values of 314.14 HV10 in the XY plane and 323.19 HV10 in the XZ plane, indicating a significantly softer microstructure. For the SDSS 40 μm LT sample, hardness was similar in both planes (424.46 HV10 XY and 425.23 HV10 XZ), and the overlapping standard deviations (SD) (11.05 and 17.0 HV10) suggest minimal mechanical anisotropy. The SDSS 80 μm LT sample, which exhibited the third-highest hardness values overall, showed slightly higher hardness in the XZ plane (384.62 HV10) compared to the XY (368.63 HV10). The variability between planes of the 80 μm LT sample was comparable to that observed for the SDSS 40 μm LT sample, with standard deviations of 16.74 HV10 (XY) and 9.41 HV10 (XZ). The SDSS-Remelting sample showed higher hardness in the XZ plane compared to the XY plane as well, with relatively low standard deviations (~9 HV10), reflecting uniform hardness distribution per plane. The SDSS-Blend sample demonstrated slightly higher hardness in the XZ plane (323.19 HV10) compared to the XY (314.14 HV10), with even lower standard deviations (~6–7 HV10) in comparison with the SDSS 40 and 80 μm LTs and SDSS-Remelting, showing better uniformity across planes.

### 3.4. Phase-Separated Nanomechanical Properties

Table 6 summarizes the number of indents assigned to the ferrite and austenite phases. The ferrite phase exhibited a higher number of indents than austenite. Because the γ phase in the SDSS-Blend occurred predominantly as grain-boundary/Widmanstätten plates/films, the accessible area for phase-pure indents was inherently smaller than for δ. Therefore, variations in indent counts among phases arise from differences in their size, morphology, and distribution within the microstructure, rather than from preferential placement. The nanomechanical properties of each phase are presented in Table 7. No notable differences were observed between the ferritic and austenitic phases in the SDSS-Blend sample with respect to any of the investigated mechanical properties, namely the reduced modulus (E_r_), hardness (H), or plasticity ratios (h_c_/h_max_, H/E_r_). The average values of the nanomechanical properties were similar, along with their standard deviations. The elastic and plastic work of both phases (Table 8), assessed in conditions with and without pop-in correction, followed similar trends consistent with the previously discussed nanomechanical properties. A similar relationship between the two phases was observed for the total work, defined as the sum of the elastic- and plastic-energy contributions. Overall, both phases demonstrated comparable nano-scale toughness (energy absorption capacity).

## 4. Discussion

### 4.1. Processing and Microstructure

The process-parameter-optimization study conducted for both alloys and processing conditions successfully identified parameters that yielded high-density samples. The SDSS 80 µm LT and SDSS-Blend approaches attained optimal relative densities exceeding 99.9% (Figure 5). In comparison, the remelting strategy yielded lower densification, with the maximum relative density approaching 98% (Figure 5). The SDSS 80 µm LT cases (approximately 53–59 J/mm^3^) were positioned near the low energy threshold; nevertheless, porosity remained as gas-type without Lack of Fusion (LoF) defects, indicating that the selected parameters ensured adequate overlap and penetration, even at greater layer thicknesses. This pattern of defect evolution is consistent with process maps and mechanistic studies that correlate LoF with insufficient overlap, and keyhole/gas porosity with higher local energy and melt-pool instability [37].

These findings also corroborate reports identifying hatch spacing as a primary factor influencing overlap [41]. In Super Duplex Stainless Steel SDSS 2507 processed by Laser Powder Bed Fusion LPBF, the pore type scales systematically with volumetric energy density [42]: at low energy density, insufficient melt-pool penetration and overlap across the tracks/layers produce LoF pores; moving into a moderate energy-density window (≈68–127 J/mm^3^), melt-pool geometry and overlap are adequate and densities ≥ 99.6% are achieved; at higher energy density (>127 J/mm^3^), excessive local energy drives keyhole/gas porosity (rounded pores) as melt-pool stability degrades (vapor depression and collapse). This trend, and the optimized parameter set (laser power–scan speed–hatch spacing) reported to reach ~99.96% density, was mapped explicitly by Mulhi et al. [42] for 2507 over a broad energy density span (22–429 J/mm^3^), identifying the high-densification zone and clarifying the transition from LoF to the optimum and keyhole regimes with increasing energy density. Importantly, while energy density is a useful first-order guide, identical energy densities achieved with different power–speed–hatch combinations can yield different melt-pool physics and defect outcomes, so explicit tuning of laser power, scanning speed, layer thickness for width/depth, and overlap is essential to remain in conduction mode and avoid keyhole instability [43].

With the low-energy remelting parameters employed in this study, the SDSS-Remelting sample exhibited LoF voids at the highest total Volumetric Energy Density (VED) (79.2 J/mm^3^, RM_4). This can be rationalized by the low VED of the remelting pass (20.8 J/mm^3^), which produced a shallow reheating melt pool that is insufficient to penetrate bead valleys and re-bond track edges across the 0.10 mm hatch and interlayer interfaces; consequently, LoF propagated through the section and remained visible. This interpretation accords with LPBF defect maps in stainless steels, also mentioned in previous sections, showing that incomplete overlap and inadequate remelt depth govern planar, boundary-parallel LoF, whereas excess energy promotes spherical gas/keyhole porosity [43]. For SDSS 2507, optimized single-scan (with powder) windows that minimize LoF typically sit near 68–127 J/mm^3^ [42]; operating a second scan (without powder) at a substantially lower energy density, as in this study, can probably reduce surface roughness (not the aim of the current study), yet fails to heal sub-surface underlap, explaining the prevalence of LoF in both the XY and XZ planes despite the high total energy input.

Microstructurally, the as-built SDSS 40 µm LT, SDSS-Remelting, and SDSS 80 µm LT samples were predominantly δ-ferritic, exhibiting thin Grain Boundary Austenite (GBA) in the former two cases and coarser GBA in the 80 µm LT sample (Figure 6). Zhang et al. [44] showed that thicker powder layers promote slower cooling and, consequently, coarser grain structures. Increasing the layer thickness can alter the LPBF thermal history toward slower effective cooling, and thereby promote austenite formation in duplex/super-duplex steels. A thicker powder layer increases the thermal resistance between the melt pool and the solid heat sink, and typically enlarges the melt-pool volume, both of which reduce heat extraction and extend the residence time in the ≈800–1200 °C window, where δ-ferrite transforms to γ-austenite by diffusion of interstitials and partitioning of Ni/Cr/Mo; this favors grain-boundary γ films and intragranular (often Widmanstätten-type) γ in the as-built structure [45]. The mechanistic link between cooling rate and γ morphology and fraction is well-established for LPBF-processed Duplex Stainless Steel (DSS) and SDSS. As-built microstructures are dominantly δ-ferritic, and slower thermal trajectories (longer local dwell times or reduced heat flux) increase the amount of boundary-nucleated and plate-like γ, whereas faster cooling suppresses Widmanstätten growth (observed, for example, with build-orientation-induced changes in track length and heat flow) [6,46].

In the SDSS–SS316L blend sample, the compositional change significantly increased the austenite phase fraction and additionally promoted the formation of fine, plate-like Widmanstätten-type intragranular γ. This indicated that SS316L addition enhanced γ stabilization in the as-built condition and favored refined plate/film morphologies. Based on quantitative microstructural phase analysis, modification of the alloy’s chemical composition led to a significant increase in austenite content, with the SDSS-Blend exhibiting an approximately 50/50 austenite–ferrite microstructure in the as-built condition (Figure 6 and Figure 7). Blending led to a rise in the major austenite-stabilizing element nickel, from 8.0 wt% in SDSS to 10.3 wt% in the SDSS-Blend, and a reduction in the major ferrite-stabilizing element chromium, from 24.7 wt% in SDSS to 20.9 wt% in the SDSS-Blend (Table 1). In the SDSS-Blend, the presence and higher fraction of Widmanstätten-type austenite is mainly composition-driven. These compositional changes favor the stabilization of austenite over ferrite, resulting in the observed higher austenite fraction in the SDSS-Blend. Cui et al. [25] also observed that a precise 50/50 ratio resulted in 20–30% austenite content, depending on the quantification method, which is significantly lower in comparison to the austenite volume fraction achieved in the SDSS-Blend. This is attributed to the minor variations in powder composition, processing, and mixing parameters. Shoji Aota et al. [47] mentioned that proper powder mixing and optimized laser parameters enable better alloy homogenization and more control over phase formation. The study highlights that poor powder mixing or inappropriate process conditions can cause heterogeneous microstructures and phase distributions [47]. Our observation of increased as-built γ content in the SDSS-Blend also aligns with previous LPBF blend studies of DSS/SDSS–316L, which report higher γ fractions than in monolithic SDSS [26]. The plate morphology is consistent with the duplex transformation sequence from grain-boundary γ films to intragranular plates (Widmanstätten), advancing along the {100}δ∥{111}γ planes when local dwell permits, according to previous studies [6,46]. Together, the elevated γ stability from the blend and a process window that avoided melt-pool instability provided a coherent basis for the Widmanstätten-rich, near-duplex microstructure observed.

Regarding the SDSS-Remelting sample, the configuration using a higher VED in the first scan followed by a lower VED in the second, although within the optimum relative-density parameters, did not significantly slow the solidification of the remelted skin. The effective cooling rate remained high because the small melt-pool volume and short dwell dominate the local thermal history. On the other hand, the preheating from the first scan was insufficient to hold the sub-surface in the 800−1200 °C window needed for the δ→γ transformation. This reading is consistent with melt-pool thermal measurements showing that the cooling rate is set by the melt-pool size and energy input, and with layer-by-layer remelting studies where low-energy rescans maintain high temperature gradients and rapid solidification in the skin. It also aligns with duplex/SDSS observations that laser-remelted surfaces resolidify to near-fully ferritic layers with Cr_2_N (an indication of fast cooling). Thus, short rescans of this type do not effectively provide enough reheating to form austenite beneath the remelted layer [46,47,48,49,50].

With regards to the composition of the phases, in 40 and 80 μm LT SDSS samples, no compositional variation could be identified through Energy Dispersive X-Ray Spectroscopy (EDS) in the ferrite/austenite phases, whereas in the SDSS-Remelting sample, both phases exhibited high Mo and reduced Cr contents (Table 4). The observed reduction in chromium content in both phases in the SDSS-Remelting microstructure may be attributed to the increased precipitation of chromium nitrides during remelting, suggesting an increased cooling rate. Chromium is effectively sequestered within these precipitates, leading to chromium depletion in the surrounding matrix phases [51]. This elemental redistribution contributes to the relative enrichment of molybdenum within the phases [52]. Furthermore, the phase elemental composition in the SDSS-Blend sample differed from those of the SDSS 40 μm, 80 μm layer LT, and SDSS-Remelting samples due to its different alloy chemical composition.

### 4.2. Macro-Hardness Variations

Across the four conditions (Figure 8, Table 5), the SDSS-Remelting specimen showed the highest hardness (XY: 459.7 ± 9.2 HV10; XZ: 468.6 ± 9.3 HV10), followed by SDSS 40 µm LT (XY: 424.5 ± 11.1; XZ: 425.2 ± 17.0 HV10) and SDSS 80 µm LT (XY: 368.6 ± 16.7; XZ: 384.6 ± 9.4 HV10); the SDSS-Blend condition exhibited the lowest values (XY: 314.1 ± 6.2; XZ: 323.2 ± 6.9 HV10). The observed standard deviations (>10 HV10 in several cases) were accounted for by local microstructural variability (grain-size dispersion, residual stress, nitride-rich regions) and indent positioning [53,54]. Plane-to-plane differences remained modest, indicating limited macro-scale anisotropy for the indentation footprint employed, in agreement with prior LPBF duplex/SDSS studies that reported small XY–XZ hardness gaps, primarily attributable to grain orientation and local heterogeneity [55].

The higher hardness of the remelted surface was explained by its thermal history. The remelting pass was applied at a lower VED than the primary scan, which created a shallow, transient melt pool and maintained a high effective cooling rate in the remelted skin. This favored grain refinement (Hall–Petch strengthening) [56] and limited the development of grain-boundary γ in the remelted layer, both consistent with the observed hardness increase. In situ thermography and modeling studies showed that cooling rate in LPBF depended strongly on the melt-pool size and energy input; layer-by-layer remelting at reduced energy typically retained or raised the temperature gradient and local cooling in the skin [15]. In SDSS, rapid cooling could also promote Cr_2_N precipitation within ferrite, which increases ferrite hardness and could marginally elevate bulk hardness under such conditions [57].

The systematic changes observed with increasing layer thickness can be attributed to corresponding changes in cooling rate, indicating a cooling-rate-controlled mechanism; hardness decreased from ~425 HV10 at 40 µm to ~369–385 HV10 at 80 µm, consistent with the lower effective cooling rate at the thicker layer, grain coarsening, and extended time for grain-boundary austenite to nucleate and grow. Gor et al. [58] reported that hardness decreases with increased layer thickness due to coarsening of grains and reduced cooling rates during solidification. This interpretation aligned with the Hall–Petch relationship (finer grains yield higher hardness) and with established LPBF observations that increasing layer thickness promotes coarser microstructures and thus lower hardness [56].

The SDSS-Blend sample exhibited the lowest HV10 values. This outcome was consistent with its γ-stabilized composition (higher Ni-equivalent and lower Cr/Mo/N than SDSS) [59], which increased the as-built austenite fraction and reduced ferrite-based solid-solution strengthening [60] effects that were reported in LPBF-processed DSS/SDSS–316L blends [26] and matched the present microstructural observations.

The 98% relative density of the SDSS-Remelting sample did not significantly affect hardness. Studies [61,62,63] have shown that for LPBF parts with a relative density of ~98%, micro-hardness and macro-hardness values are generally unaffected. However, the negative impact of porosity becomes more pronounced if the porosity exceeds 2%, if pores are interconnected, or if hardness indentations coincide with pore locations. In this study, all indentations were performed in pore-free regions, avoiding any influence of porosity on the measured hardness.

### 4.3. Nanomechanical Properties of Duplex Microstructure

Nanoindentation mapped phase-resolved H, E_r_, H/E_r_, and h_c_/h_max_ in the as-built SDSS-Blend; the austenite and ferrite phases showed statistically comparable responses (H ≈ 3.62 vs. 3.64 GPa, E_r_ ≈ 160 vs. 162 GPa, H/E_r_ ≈ 0.02, h_c_/h_max_ ≈ 0.91 for both), indicating no meaningful phase-to-phase contrast in local stiffness or hardness, and the plasticity indices (H/E_r_, h_c_/h_max_) fell in the same range for γ and δ, implying similar indentation ductility at the grain scale; these indices have been widely used as comparative measures of local plasticity in duplex stainless steels and showed strong microstructural sensitivity in high-throughput nanoindentation– Electron Backscatter Diffraction (EBSD) studies (higher values of h_c_/h_max_ and H/E_r_ point to brittle behavior, while lower h_c_/h_max_ and H/E_r_ values indicate ductile behavior) [33]. The literature on phase ordering is not uniform: some reports found ferrite harder/stiffer than austenite (e.g., MFM-guided nanoindentation), whereas others showed austenite as harder, particularly when the nitrogen enrichment in γ was significant or with negligible differences when test load, orientation, and substrate effects were rigorously controlled; such divergence has been attributed to chemistry (Ni/Cr/Mo/N partitioning), thermal history, hydrogen charging, and mapping methodology. Zhang et al. [64] measured the phase nanomechanical properties of DSS in the annealed and water-quenched state and found that ferrite has higher nano-hardness than austenite. Gadelrab et al. [65] also concluded that the ferrite is the stronger phase compared to austenite, with higher elastic modulus and nano-hardness values. The relatively low nitrogen content of the alloy limited planar glide in austenite, reducing its strengthening during deformation, and thereby explaining why austenite did not appear harder and stiffer than ferrite [65]. Tao et al. [66] have found that the nano-hardness of ferrite and austenite is not too different without hydrogen charging in the annealed condition of as-rolled 2205 DSS alloy. Queguineur et al. [67] have shown that austenite presents a higher nano-hardness and elastic modulus in comparison to ferrite in both DSS samples produced with low- and high-heat input, respectively, through wire-arc additive manufacturing. The higher hardness of the austenite phase in this study can be attributed to its FCC structure with low stacking fault energy, which promotes dislocation multiplication and uniform dislocation distribution. The authors stated that the observed differences in hardness between the phases can be attributed to the higher nitrogen content in the austenitic phase, which is a planar-glide-deformation promoter.

Thus, the differences in nanomechanical properties between the two phases can be influenced by several factors, including solid-solution strengthening from the alloying elements and their distribution within each phase, as well as the crystallographic orientation, grain size of the phases [59,60], the strengthening of austenite during deformation, and its nitrogen content.

In the present alloy, Table 4 showed similar elemental partitioning between phases, which rationalized the near-identical H and E_r_; moreover, the rapid LPBF cooling likely limited N transfer to γ and may have consumed N as Cr_2_N in δ—both effects flattening phase differences at the grain scale, consistent with observations of quenched-in Cr_2_N in SDSS 2507 and laser-remelted DSS under fast cooling [68]. Processing dependence further supported this view [69]: in conventionally processed duplex steels, hot-forging/work-hardening elevated phase hardness (sometimes favoring γ formation over δ), while as-cast/annealed states often showed smaller or reversed gaps, underscoring that deformation history and nitrogen availability govern the phase ordering more than crystallography alone. The phase-equivalent nanomechanics of the SDSS-Blend indicated that its lower macro-hardness (HV10; Section 4.2) did not originate from a softer constituent phase; rather, it reflected bulk compositional effects combining a γ-stabilized chemistry (higher Ni-equivalent, reduced Cr/Mo/N) that increased the austenite fraction and diminished ferritic solid-solution strengthening with rapid LPBF cooling and Cr_2_N precipitation in δ, further homogenizing the phase-level mechanical response.

By computing the area under the curve (AUC), the indentation work for each phase in the SDSS-Blend has been effectively quantified. The loading AUC provides the total energy input, whose partitioning into elastic versus plastic components offers insights into material behavior. The plastic portion of the work is a measure of energy dissipated in permanent deformation, and is thus directly related to the material’s capacity to absorb energy. This approach is supported by standard nanoindentation analysis techniques and has been used in the literature to correlate with mechanical properties and deformation characteristics [70]. In our results, the ferrite and austenite phases showed nearly identical AUC values, indicating similar hardness and local toughness in the as-built state. This finding is noteworthy because in many multi-phase alloys (like dual-phase steels or cast irons), one phase might be harder and less tough than the other, leading to different indentation toughness [71,72,73]. Here, however, the ferritic and austenitic regions of the SDSS-Blend behaved almost identically in how they absorbed deformation energy. In the unmodified dataset, the mean loading AUCs were 347.0 pJ (ferrite) and 348.1 pJ (austenite), with corresponding unloading AUCs of 46.7 pJ and 45.8 pJ, respectively. The resulting total AUCs, indicative of energy dissipated via plastic deformation, were 300.2 pJ (ferrite) and 302.3 pJ (austenite). After truncation of pop-ins, the loading AUCs slightly decreased to 345.8 pJ (ferrite) and 346.1 pJ (austenite), while total AUCs remained consistent at 299.4 pJ and 300.1 pJ, respectively. Across both datasets, standard deviations exhibited minimal variation, underscoring the statistical robustness of the measurements. The close alignment of statistical measures between phases confirmed that neither phase is significantly more deformation-resistant or energy-absorbing than the other. Finally, by addressing pop-in artifacts, either by including popped-in curves or truncating the pop-in regions, the confidence in these metrics is improved. In this case, both methods yielded consistent results, implying that pop-ins did not materially alter the conclusion.

Cheng et al. [73] established a relationship between the ratio of plastic to total work (Wp/Wt) and the hardness-to-modulus ratio (H/E_r_), showing that materials with higher H/Er tend to exhibit lower plastic work fractions. Similarly, Yamamoto et al. [70] demonstrated that the ratios of plastic to elastic work (Wp/We) are thermodynamically constrained and correlate with the mechanical energy balance during indentation. In their study, the convergence of Wp/We toward unity at high H/E_r_ values was interpreted as a limit beyond which further plastic deformation becomes energetically unfavorable.

In the present study, the mean total AUC values and their narrow standard deviations across all phases suggest that both ferrite and austenite in SDSS-Blend possessed comparable resistance to plastic deformation and similar capacities for energy absorption. The minimal differences observed between the direct and truncated datasets further reinforce the robustness of this behavior. The truncation of pop-ins—abrupt displacement bursts typically associated with incipient plasticity or dislocation nucleation—did not significantly alter the statistical outcomes. This indicates that the material’s overall response was not dominated by early-stage instabilities and that the energy metrics are representative of the intrinsic phase behavior.

The presence of pop-ins, while potentially disruptive to AUC calculations, was effectively managed through the dual approach of discarding or truncating affected curves. The consistency of results between these methods suggests that the influence of pop-ins on the overall energy metrics was limited. This aligns with recent findings by Kossman et al. [74], who emphasized the importance of identifying and managing pop-in events to ensure accurate mechanical property extraction from nanoindentation data.

Overall, the results support the conclusion that the SDSS-Blend exhibited a high degree of mechanical isotropy, with both the ferritic and austenitic phases demonstrating similar toughness and deformation characteristics. This uniformity is advantageous for applications requiring consistent mechanical performance across microstructural features, and it reflects the effectiveness of the processing conditions in homogenizing the mechanical response of the alloy. This phase-independent energy-absorption behavior observed in the SDSS-Blend aligns well with the mechanical isotropy requirements of advanced structural applications, where uniform stress distribution and predictable deformation responses are critical. The near-identical values of H, E_r_, H/E_r_, h_c_/h_max_, and indentation energy across ferritic and austenitic regions suggest that the microstructure can accommodate mechanical loads without preferential localization of strain or failure. Such isotropic performance is particularly advantageous in components subjected to complex loading conditions—such as in aerospace, biomedical implants, or energy systems—where consistent mechanical integrity across all directions and microstructural domains is essential for reliability and longevity.

## 5. Conclusions

This study systematically investigated the influence of two processing strategies—layer-thickness adjustment and laser remelting—and one compositional modification approach (powder blending with austenitic stainless steel) on the microstructure and mechanical properties of Super Duplex Stainless Steel (SDSS) alloys fabricated via Laser Powder Bed Fusion (LPBF). The primary objective was to promote austenite formation and achieve a duplex microstructure while elucidating the underlying mechanisms governing phase distribution and mechanical behavior.

Key microstructural findings revealed that compositional modification through powder blending with Stainless Steel SS316L was the most effective strategy for balancing phase fractions, yielding a duplex microstructure with nearly equal proportions of austenite and ferrite (~45–55% austenite and ~55–45% ferrite). In contrast, increasing layer thickness and applying laser remelting did not significantly alter the overall austenite content relative to the reference SDSS processed at a 40 μm layer thickness.

Mechanical property analysis underscored the critical role of microstructure evolution. The remelting strategy markedly increased macro-hardness (~460 HV10) over the reference 40 μm layer thickness (~425 HV10), attributable to reduced grain boundary austenite, finer grain structure, enhanced chromium nitride precipitation, and more pronounced elemental redistribution between phases—effects driven by higher cooling rates. Conversely, slower cooling associated with increased layer thickness promoted grain coarsening and well-developed austenite at the ferrite boundaries, resulting in reduced macro-hardness (~377 HV10). The SDSS-Blend alloy exhibited the lowest macro-hardness (~320 HV10), a consequence of its modified chemical composition that diminished solid-solution strengthening despite the nanomechanical equivalence of its austenite and ferrite phases.

These results demonstrate that optimizing duplex microstructure and mechanical properties requires careful integration of both processing parameters and targeted compositional adjustments. For practitioners and researchers seeking to tailor LPBF-processed SDSS alloys, the study provides clear guidance: compositional modification is essential for achieving balanced duplex microstructures, while processing strategies such as remelting and layer-thickness control should be leveraged to fine-tune grain size and hardness. The interplay between cooling rate, phase distribution, and alloy chemistry must be strategically managed to realize desired property profiles.

Future work should explore the effects of alternative alloying additions, extended heat treatments, and advanced in situ characterization techniques to further elucidate phase evolution and mechanical performance. Systematic studies on the relationship between process-induced microstructural features and service behavior, including corrosion resistance and toughness, will be vital for the deployment of LPBF-processed SDSS alloys in demanding engineering applications.

## Figures and Tables

**Table 1:** Remelting strategy DoE (SDSS-Remelting) parameters for SDSS processed through LPBF.

		Scan 1			Scan 2 (Remelting)			
Alloys	No. of Sample	Laser Power (W)	Scan Speed (mm/s)	VED (J/mm^3^)	Laser Power (W)	Scan Speed (mm/s)	VED (J/mm^3^)	Total VED (J/mm^3^)
SDSS	RM_1	200	1200	41.67	100	800	31.25	72.92
	RM_2	220	1200	45.83	120	1200	25	70.83
	RM_3	250	1200	52.08	50	600	20.83	72.92
	RM_4	280	1200	58.33	50	600	20.83	79.17

**Table 2:** The 80 μm layer-thickness DoE (SDSS 80 μm LT) parameters for SDSS processed through LPBF.

Alloys	No. of Sample	Laser Power (W)	Scan Speed (mm/s)	Hatch Distance (mm)	VED (J/mm^3^)
SDSS	80 μm_1	308	803	0.09	53.27
	80 μm_2	337	718	0.1	58.67
	80 μm_3	388	909	0.09	59.28
	80 μm_4	336	797	0.1	52.70
	80 μm_5	439	929	0.1	59.07
	80 μm_6	384	824	0.11	52.96
	80 μm_7	219	547	0.09	55.61
	80 μm_8	85	184	0.1	57.75

**Table 3:** DoE parameters for SDSS-Blend processed through LPBF.

Alloys	No. of Sample	Laser Power (W)	Scan Speed (mm/s)	Hatch Distance (mm)	VED (J/mm^3^)
SDSS-Blend	SDSS-Blend_1	305	1174	0.11	59.04
	SDSS-Blend_2	247	756	0.10	81.68

**Table 4:** Indicative EDS phase elemental compositions for each alloy and condition.

Alloy & Condition	Austenite Composition wt%		Ferrite Composition wt%	
SDSS 40 μm LT	Cr	24.6	Cr	25.8
	Fe	64.0	Fe	61.9
	Ni	7.1	Ni	6.3
	Mo	4.3	Mo	6.0
SDSS 80 μm LT	Cr	25.8	Cr	26.4
	Fe	63.5	Fe	61.6
	Ni	6.4	Ni	6.2
	Mo	4.4	Mo	5.8
SDSS-Remelting	Cr	23.1	Cr	23.6
	Fe	64.3	Fe	62.7
	Ni	6.3	Ni	6.1
	Mo	6.3	Mo	7.6
SDSS-Blend	Cr	21.3	Cr	21.3
	Fe	65.0	Fe	63.0
	Ni	10.1	Ni	10.4
	Mo	3.6	Mo	5.3

**Table 5:** Summarized macro-hardness values for each alloy and condition.

Alloy & Condition	XY Plane	XZ Plane
SDSS 40 μm LT	424.46 ± 11.05 HV10	425.23 ± 17.0 HV10
SDSS 80 μm LT	368.63 ± 16.74 HV10	384.62 ± 9.41 HV10
SDSS-Remelting	459.73 ± 9.24 HV10	468.57 ± 9.28 HV10
SDSS-Blend	314.14 ± 6.16 HV10	323.19 ± 6.90 HV10

**Table 6:** Distribution of nanoindentation indents assigned exclusively to the ferritic and austenitic phases for the SDSS-Blend alloy.

Phases	Indents
Ferrite	141
Austenite	22

**Table 7:** Phase-separated nanomechanical properties, namely the reduced modulus (E_r_), nano-hardness (H), normalized pile-up-to-sink-in height (h_c_/h_max_), and the plasticity index (H/E_r_), concerning the SDSS-Blend alloy.

Phase	E_r_ (GPa)	H (GPa)	h_c_/h_max_	H/E_r_
Ferrite	161.98 ± 6.49	3.64 ± 0.18	0.91 ± 0.01	0.02 ± 0.01
Austenite	159.79 ± 5.11	3.62 ± 0.12	0.91 ± 0.01	0.02 ± 0.01

**Table 8:** Statistical summary of the area under the nanoindentation load–displacement curves (AUC) for the loading, unloading, and total energy components in SDSS-Blend, computed (**a**) without pop-in correction, and (**b**) following truncation of pop-in events. Values are reported in picojoules (pJ) for each phase (ferrite, austenite), including mean and standard deviation.

(**a**)		
**Curve Area**	**Phase**	**Mean**
Loading (plastic) (pJ)	Ferrite	347.0 ± 17.0
	Austenite	348.1 ± 14.2
Unloading (elastic) (pJ)	Ferrite	46.7 ± 4.5
	Austenite	45.8 ± 4.3
Total (plastic + elastic) (pJ)	Ferrite	300.2 ± 14.1
	Austenite	302.3 ± 11.5
(**b**)		
**Curve Area**	**Phase**	**Mean**
Loading (plastic) (pJ)	Ferrite	345.8 ± 16.5
	Austenite	346.1 ± 11.7
Unloading (elastic) (pJ)	Ferrite	46.3 ± 4.8
	Austenite	46.1 ± 2.6
Total (plastic + elastic) (pJ)	Ferrite	299.4 ± 13.3
	Austenite	300.1 ± 10.3

## Data Availability

The data presented in this study are available upon request from the corresponding author. The data are not publicly available due to privacy restrictions.
